# Deceased donor kidney transplanted in childhood functioning well after 52 years

**DOI:** 10.1007/s00467-023-05901-5

**Published:** 2023-03-16

**Authors:** Giuseppina Spartà, Karine Hadaya, Luc Paunier, Eric Girardin, Ernst Leumann

**Affiliations:** 1 Pediatric Nephrology Unit, University Children’s Hospital, Zurich, Switzerland; 2 Service of Nephrology and Hypertension, Geneva University Hospitals, Clinique Des Grangettes-Hirslanden, Geneva, Switzerland; 3 Pediatric Nephrology Unit, Department of Pediatrics, Geneva University Hospital, Geneva, Switzerland

**Keywords:** Paediatric, Kidney transplantation, Haemolytic uraemic syndrome, Dialysis, Long-term survival, Child

## Abstract

**Background:**

Kidney transplantation in children in 1970 was considered by many to be unethical, as long-term survival was minimal. It was therefore risky at the time to offer transplantation to a child.

**Case diagnosis/treatment:**

A 6-year-old boy with kidney failure due to haemolytic uraemic syndrome received 4 months of intermittent peritoneal dialysis followed by 6 months of haemodialysis until at 6 years and 10 months, he underwent bilateral nephrectomy and received a kidney transplant from a deceased 18-year-old donor. Despite moderate long-term immunosuppression of prednisone (20 mg/48 h) and azathioprine (62.5 mg/day), at the last visit in September 2022, he was well, normotrophic, with a serum creatinine of 157 µmol/l (*eGFR* 41 ml/min/1.73 m^2^) and no haematuria, proteinuria or hypertension. Except for benign skin lesions due to azathioprine, and undergoing an aortic valve replacement and an aortic aneurysm repair in adulthood, the now 58-year-old man has had no major complications.

**Conclusions:**

We speculate that stable and unmodified immunosuppressive therapy, started before the era of calcineurin inhibitors, the lack of significant rejection episodes, the absence of donor-specific antibodies, and the young donor age have contributed to maintaining exceptional long-term kidney transplant survival. Luck, a robust health system and an adherent patient are also important. To the best of our knowledge, this is the longest functioning kidney transplant from a deceased donor performed in a child worldwide. Despite its risky nature at the time, this transplant paved the way for others.

## Introduction

Treatment of kidney failure with haemodialysis and kidney transplantation in children began in the USA in the late 1960s, with reasonable results despite many unresolved problems [1–5]. Kidney replacement therapy in Europe was largely limited to selected adults. Scepticism concerning transplantation in children was based on anticipated severe growth failure (due to uraemia or immunosuppression with prednisone), disturbed sexual maturation and psychosocial problems. Nevertheless, it was offered to a 6-year-old boy, who after 10 months of dialysis received a kidney from a deceased young adult donor in December 1970. Despite the suboptimal immunosuppression available, the transplantation proved highly successful, and the kidney and the patient are doing well after 52 years.

## Case report

**Fig. 1:**
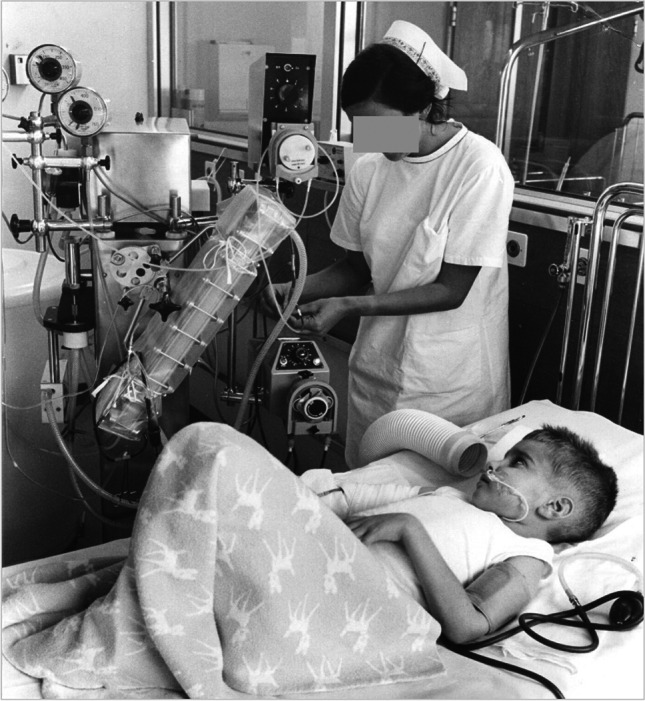
The 6½-year-old patient on haemodialysis in 1970. Not shown is the tank containing 240-l of dialysate. A 6-plate dialyser was used (reduced surface area for a child). Ultrafiltration was very limited; the child had a severe fluid restriction of 500 ml/day

The patient, a previously healthy boy of Swiss origin, was hospitalized at the Geneva University Children’s Hospital at the age of 5 years and 10 months with haemolytic uraemic syndrome (HUS). Intermittent weekly peritoneal dialysis was initially required beginning in February 1970 as his kidneys did not recover. On June 15, 1970, the boy was transferred to the Zurich University Children’s Hospital for haemodialysis in anticipation of a deceased donor kidney transplant. The primary goal of switching to haemodialysis was to improve his poor nutritional status (height 116 cm, 25 percentile, weight 16.3 kg, < 3rd percentile). Haemodialysis was performed for 5 h, 3 times weekly for 6 months, using a Gambro 6 plate (paediatric) dialyser (Fig. 1), through a Scribner shunt. Monthly leucocyte-poor packed red cell transfusions were required to maintain his haematocrit above 17%, as erythropoietin was not yet available. Challenges arose due to repeated clotting and an infection of the Scribner shunt. Strict fluid restriction was necessary because of anuria and the limited ultrafiltration capacity of the dialyser. Nevertheless, his overall status markedly improved.

Kidney transplantation was performed by the late Dr. Felix Largiadèr at the Zurich University Hospital on December 15, 1970. The patient was 6 years and 10 months old with a dry weight of 18.4 kg (< 3rd percentile). The 18-year-old donor had suffered brain injury. Histocompatibility testing revealed an identical blood group (A) and a negative crossmatch. HLA matching in retrospect is unclear, with 1 or 2 possible known mismatches [6]. Detailed HLA status of the donor is no longer available.

Bilateral nephrectomy was performed in the recipient (standard in Zurich at the time), and the graft was inserted into the right renal fossa. The two donor renal arteries were anastomosed to the aorta and the renal vein to the vena cava. Urine output started immediately. Initial immunosuppression consisted of methylprednisolone followed by prednisone (15 mg/day), azathioprine (50 mg) and 26 days of 5 ml of ALG (antilymphocyte globulin, 12.5 mg/kg/day). A mild rejection episode, diagnosed clinically with fever, tenderness over the graft and a 60% rise in serum creatinine at 2 weeks after transplantation, responded well to an increase in prednisone to 60 mg/day followed by a rapid decrease to 30 mg/day and doubling of ALG to 10 ml/day. A second mild rejection episode was clinically suspected due to tenderness over the graft despite stable kidney function at 4 weeks after transplantation. Prednisone was again increased to 60 mg and then reduced at 3-day intervals to 45 mg and 30 mg. The boy was discharged 8-week post-transplant on daily prednisone 30 mg (later slowly reduced to 15 mg) and azathioprine 37.5/50 mg on alternate days. As expected, he developed Cushing syndrome. At the age of 8 years, daily prednisone was therefore switched to alternate day (20 mg), permitting growth along the 3rd percentile. The patient attended regular school and leads a normal adult life.

As of September 2022, the patient is well, height 168 cm (expected height on 50th percentile was 177 cm), weight 52 kg, serum creatinine 157 µmol/l and eGFR (CKD-EPI) 41 ml/min/1.73 m^2^. He has no haematuria, albuminuria or proteinuria, no hypertension, no lipid abnormalities and no diabetes. Immunosuppression still consists of prednisone (20 mg/48 h) and azathioprine (62.5 mg/day). Further donor HLA typing was attempted from the recipient’s urine without success. Crucially, he has no anti-HLA antibodies, except an anti-DP1 at low mean fluorescence intensity (MFI), therefore no significant donor-specific antibodies. Assessment of tolerance is not possible. Genetic analysis showed a homozygous CFHR3/CFHR deletion associated with atypical HUS (aHUS). He never experienced disease recurrence. Non-kidney complications have included surgical repair of an aneurysm of the ascending aorta at age 45 years and prosthetic aortic valve replacement for severe aortic insufficiency at age 57 years. Due to azathioprine, he has had benign keratoses.

## Discussion

The late 1960s was a pioneering time for dialysis and kidney transplantation, particularly in children [1–5]. Long-term haemodialysis and kidney transplantation in young paediatric patients had only very rarely been performed in Europe. In Switzerland, there was widespread consensus that only patients aged 15 to 50 years should be offered kidney replacement therapy [6]. Early short-term results of kidney transplantation in younger patients performed in the USA had, however, shown promising results [1–3, 5].

The senior author (E. L.) had received training in 1969 in San Francisco with a view to introducing kidney replacement therapy for children in Switzerland. Long-term haemodialysis could only be offered once the transplant surgeon agreed to accept a young child for deceased donor transplantation. Living donor transplantation was not regarded as acceptable at the time because 1-year graft survival was < 50% [3]. The child was failing to thrive on intermittent PD, without other options in Switzerland. The decision was therefore taken to list him for a deceased donor transplant in Zurich, requiring a switch to haemodialysis to first optimize his condition.

Scepticism, however, arose from several sides. The boy’s parents had to face the dilemma of the very limited perspective offered by dialysis and the unknowns of transplantation. A nephrologist elsewhere advised the family against transplantation. In addition, several colleagues within the hospital criticized this treatment. The engagement of the nurses, who first had to be trained in haemodialysis (see Fig. 1) and then in transplant care was essential, as was that of the other team members, especially the dietitian and the kindergarten teacher, who provided psychosocial support. The child suffered most from the severe fluid restriction. It was helpful that he could spend weekends between dialysis sessions at home in Geneva, awaiting the call for transplantation. This successful transplant encouraged continued paediatric transplantation in Switzerland.

We now know that the numerous determinants of impaired graft survival include development of donor-specific antibodies, rejection, infection, malignancy, primary disease recurrence, and low adherence to lifelong drug intake [7–10]. Despite improvements in short-term survival, this has not translated into improved long-term graft survival. The current estimated half-life for a transplanted kidney in children is only 15 years; therefore, children with kidney failure usually require more than one kidney transplant in their lifetimes [10]. Both the USA and European registry data show relatively stable graft failure rates beyond the first-year post-transplantation since the late 1980s [7–11]. In the USA, the medium-term deceased-donor graft survival rate from 1996 to 1999 was only 20% at 20 years. The steady loss of kidney function over time remains an unsolved problem. Data on 50-year graft survival after deceased donation do not exist. In fact, hardly any adult recipient of a deceased donor graft would still be alive after 50 years.

It was great luck that the donor was young, the patient was not sensitized despite the multiple transfusions, and only experienced two minor rejection episodes. The immunosuppression with prednisone and azathioprine, started before the era of calcineurin inhibitors, was not modified, following the principle of “never changing a winning horse”. The kidney was therefore spared potential calcineurin toxicity. Crucially, he still has no significant  donor-specific antibodies . Additionally, despite having aHUS as a risk factor, he did not experience any disease recurrence. Furthermore, the patient has always had access to quality medical care and medication and has been adherent all his life. All of these factors contributed to graft function which remains good at 52 years of follow-up. To the best of our knowledge, this is the longest functioning kidney transplant from a deceased donor performed in a child worldwide and is testament to the importance of robust health systems that facilitate optimal health outcomes.
